# A Comparative Study on the Wear Performance and High-Temperature Oxidation of Co-Free Cermets and Hardmetals

**DOI:** 10.3390/ma17143615

**Published:** 2024-07-22

**Authors:** Ángel Biedma, Gabriel Sánchez, María de Nicolás, Claudio Bertalan, Ralph Useldinger, Luis Llanes, Elena Gordo

**Affiliations:** 1 GTP—Department of Materials Science and Engineering, IAAB, Universidad Carlos III de Madrid, 28911 Leganés, Madrid, Spain; gabsanch@pa.uc3m.es (G.S.); mnicolas@ing.uc3m.es (M.d.N.); elena.gordo@uc3m.es (E.G.); 2 Sustainable Powder Technologies—IMDEA Materials Institute, 28906 Getafe, Madrid, Spain; 3 CERATIZIT Luxembourg S.à r.l., 8232 Mamer, Luxembourg; claudio.bertalan@ceratizit.com (C.B.); ralph.useldinger@ceratizit.com (R.U.); 4 CIEFMA—Department of Materials Science and Engineering, Universitat Politècnica de Catalunya—BarcelonaTech, 08019 Barcelona, Catalunya, Spain; 5 Barcelona Research Center in Multiscale Science and Engineering, Universitat Politècnica de Catalunya—BarcelonaTech, 08019 Barcelona, Catalunya, Spain

**Keywords:** alternative hard materials, wear response, high-temperature oxidation, mechanisms

## Abstract

The present investigation addresses the mechanical properties, wear behaviour, and high-temperature oxidation of cermets and hardmetals based on either Ti(C,N) or WC and a metal binder based on Fe15Ni or Fe15Ni10Cr. This study also includes a commercial-grade WC-Co for comparative purposes. The production of these materials involved a powder metallurgy and sinter-HIP processing route under identical conditions. It is found that WC-based materials have superior mechanical properties, including hardness, fracture toughness, transversal rupture strength (TRS), and wear response, compared to Ti(C,N)-based materials. However, the latter show better oxidation behaviour than the former. Notably, WC-FeNi exhibits a higher hardness and TRS than the commercial-grade material (an increase of 7% and 9%, respectively). The difference in wear behaviour is due to the difference in wear mechanisms. In this regard, cermets wear through a tribolayer of Ti and Fe oxides, while hardmetals primarily wear through abrasion from ploughing. Thus, hardmetals exhibit a lower coefficient of friction (COF) and wear rate than cermets. Furthermore, Ti(C,N)-based materials form a protective layer of TiO_2_, which enhances their integrity and reduces mass gain. The addition of Cr to the FeNi binder only appears to have a clear effect on the TRS of the materials.

## 1. Introduction

Hardmetals, especially WC-Co, are undisputed first-choice materials in the tooling industry. Compared to other cutting materials, their combination of ceramic and metallic phases generates excellent properties, especially in terms of their hardness–toughness ratio [1]. However, W and Co have been included in different lists of critical raw materials (CRMs) [2]. The hard metal industry depends on CRMs for the properties they provide and their industrial history. However, the industry faces supply risks and extraction challenges, which lead to the demand for alternative materials. The high and fluctuating market prices of their raw materials over time have also been a rationale for their replacement [3]. In addition, regarding health issues, Co has been catalogued as a potentially hazardous substance due to its potential genotoxic effect [4], which is even increased when combined with WC or TiC [5].

These facts have driven both the industry and the scientific community to search for alternative materials. Currently, two strategies are being pursued: the partial or complete replacement of the metal phase and the quest for a substitute for WC [6]. In terms of the first approach, extensive research has been conducted on Fe, Ni, and their alloys as potential binders due to their widespread availability, affordability, and non-toxic nature, among other advantages [7]. The second approach focuses on the potential substitution of the ceramic phase, with Ti(C,N) as a promising option due to its exceptional hardness, resistance to wear, and chemical stability [8]. Nevertheless, the significance of WC-based hardmetals and Ti(C,N)-based cermets in the current industrial landscape has been clearly established as a result of their distinct characteristics and properties, leading to a focus on each material group for specific applications [6,9,10]. Therefore, an examination of the differences between these two material groups becomes valuable when their metal binder composition and processing methods are identical.

Extensive research efforts have focused on evaluating and understanding the characteristics of wear exhibited by cermets [11,12,13]. The abrasive wear of a carbide composite is controlled by its stiffness and depends on the fraction, grain size, and properties, primarily, of the carbide phase, as well as those of the binder [14]. Furthermore, higher fracture toughness and lower coefficients of friction are associated with improved wear behaviour [15].

A study conducted by Kumar et al. [16] investigated the sliding wear characteristics of Ti(C,N)-Ni cermets under varying loads (5, 20, and 50 N). The results indicated a direct correlation between volumetric loss and the applied load. Furthermore, the experiments revealed the occurrence of abrasion and tribo-oxidation, resulting in the formation of a tribo-oxide layer primarily composed of Fe and Ti oxides. The presence of Fe in the tribo-oxide layer was attributed to the transfer of particles from the counter material. Low loads led to increased friction due to the formation of abrasive oxides of transition metals, while higher loads resulted in the formation of a protective tribo-chemical layer. In addition, the same authors [17] conducted further slip tests on Ti(C,N)-Ni cermets, incorporating various secondary carbides (WC/NbC/TaC) at different loads to investigate the influence of load and added carbides on wear behaviour. Their results revealed that, at low loads, their friction and wear characteristics were primarily influenced by the microstructural components of Ti(C,N)-Ni. In this regime, the dominant mechanism involved the formation of abrasive oxides of the transition metals from the counter material, resulting in elevated friction levels. Conversely, higher loads induced significant temperature rises, leading to the development of a tribo-chemical layer that offered protection to the surface of the Ti(C,N)-based cermet. This protective layer, irrespective of the presence of secondary carbides, effectively reduced friction and wear. In another study conducted by Onuoha et al. [18], it was reported that this tribolayer is formed through continued cyclic loading, resulting in severe mechanical attrition and significant refinement in the third-body particle size, eventually forming a homogeneous thin film in the wear track. The tribolayer has a high concentration of O, which increases with the applied load and predominantly consists of the binder constituents.

The dry sliding wear behaviour of different WC-based hardmetals has been reported in previous studies [14,15,19,20]. The results obtained by Bonny et al. [20] evidenced that the wear behaviour of WC-based materials with different binder formulations is primarily controlled by their resistance to plastic deformation, where the hardness of WC has a clear effect and the binder’s nature may also have a smaller influence.

The high-temperature exposure of cutting tools requires an investigation of their oxidation behaviour to ensure their durability. A study conducted by Gómez et al. [21] investigated the oxidation behaviour of Ti(C,N)-M2 cermets compared to a reference M2 steel at 500 °C and 800 °C in a static air atmosphere. The composite material Ti(C,N)-M2 exhibits s significantly lower mass gain after exposure to high temperatures compared to the base material M2, indicating its improved oxidation resistance. The formation of protective layers of Ti oxides prevented the generation of volatile oxides, such as WO_3_. Alvaredo et al. [22] assessed the oxidation resistance of Ti(C,N)-M2 cermets in both their sintered and heat-treated forms, building upon previous work. The formation of multilayer oxide scales was observed, comprising TiO_2_ and FeTiO_3_ protective inner layers as well as an outer layer of Fe_2_O_3_. The interlayer’s composition was found to be the primary difference between the sintered and heat-treated materials, with the sintered materials exhibiting the presence of WO_2_ and Mo_3_O oxides, while these were absent in the heat-treated ones. The study also concluded that the heat-treated material exhibited a lower mass gain compared to the sintered material. The protective behaviour of the TiO_2_ oxide layer has also been reported by Chicardi et al. [23] in (Ti,Ta)(C,N)-based cermets. Their oxidation kinetics were adjusted to a parabolic law as the oxidation rate is controlled by the inward oxygen diffusion and the outward Ti diffusion through the formed TiO_2_ layer.

The abnormal oxidation mechanism of Fe in Ti(C,N)-304SS cermets was studied by He et al. [24]. It was documented that it involved the generation of a gap in the TiO_2_/304SS interface due to volume shrinkage led by the oxidation of Ti(C,N), which acts as a rapid diffusion path for Fe atoms outward to the oxidation surface, forming Fe_2_O_3_. This abnormal behaviour is attributed to the reaction of Fe atoms with O ones in the Cr-depleted region of 304SS, which results from the rapid diffusion of Cr atoms to the surface and the formation of a protective Cr_2_O_3_ film on the binder-phase surface. In another study performed by Yang et al. [25], in a Ti(C,N)-based cermet with a Ni20Cr metallic binder, no Cr-based protective layer was formed. However, the presence of Cr led to a decrease in O_2_ for inward thermodynamic activity.

Regarding the high-temperature oxidation of cemented carbides, Basu et al. [26] studied the oxidation kinetics of WC-6Co and WC-12Co. Their study revealed that the oxidation kinetics adjusted to a linear rate law. Furthermore, their findings showed that the oxidation rate increased as the metal binder fraction decreased and with an increasing O_2_ concentration in the atmosphere.

In addition, Del Campo et al. [27] studied the isothermal oxidation of WC-based hardmetals between 450 °C and 800 °C. Their oxidation mechanism involved the formation of WO_3_, CoWO_4_, and TiO_2_ in the oxide scales, with WO_3_ having a porous structure and TiO_2_ being more compact with cracks. The proliferation of CoWO_4_ at high temperatures is related to Co diffusion, leading to a decrease in WO_3_ and an increase in TiO_2_. The formation processes of WO_3_ and TiO_2_ occur independently and in parallel, while CoWO_4_ is formed as a reaction of WO_3_ with Co oxide. The growth of the oxides remains linear, but the oxide grows at a slower rate at high temperatures due to the reduced oxygen inflow through pores and cracks.

In another study, Karimi et al. [28] examined the high-temperature oxidation characteristics of WC-FeAl and compared it with a commercial WC-Co. Their findings revealed that, within the temperature range of 700–900 °C, the WC-FeAl material exhibited the formation of FeWO_4_ and FeAlO_3_ oxides, resulting in a more compact oxide layer. Consequently, this enhanced oxidation behaviour of WC-FeAl surpassed that of the commercial material. 

Long et al. [29] studied the influence of Cr on the high-temperature oxidation behaviour of WC-based hardmetals. Their oxidation mechanism involves the selective oxidation of the metallic binder at the initial stage, followed by the oxidation of the WC hard phase to form WO_3_, and a chemical reaction between WO_3_ and metal oxides from the binder phase to form a mixed oxide layer. Cr oxide is formed inside the oxide layer while NiO is formed on the surface, providing a double layer of protection. Increasing the Cr content in the Co-Ni-Cr binder phase is a key strategy for improving the oxidation behaviour and reducing the mass gain of WC-Co-Ni-Cr cemented carbide. 

In the present study, composite materials based on a ceramic phase—Ti(C,N) or WC—and a metal binder (Fe15Ni or Fe15Ni10Cr), which were developed in previous studies [30,31], are employed. Ni and Cr were used as alloying elements to compensate for the low wettability of Fe on Ti(C,N) [30,32] and to improve the corrosion behaviour of the cermet [30,33]. These materials present competitive properties in comparison to commercial materials but with a reduced content of critical raw materials. 

The primary focus of this investigation is to deepen the study of the properties and performance of these materials under conditions close to those which they are subjected to in their end-use applications. Consequently, this research aims to make progress in exploring alternative materials that can potentially replace conventional cemented carbides. Additionally, this project seeks to accomplish the following objectives:

- Study the response to high-temperature oxidation and wear of compositions based on Ti(C,N) and WC with a Co-free metallic phase (FeNiCr).
- Assess the effect of Cr in these materials by comparing them with others using an FeNi binder phase.
- Compare the behaviour of these materials with a commercial-grade WC-Co.

This study offers new perspectives through the comparison of Co-free metallic binders (FeNiCr and FeNi) with Ti(C,N) and WC as their ceramic phase in relation to a commercial WC-Co under the same oxidation and wear conditions. This research assesses sustainable alternatives for the hardmetal industry, with implications for both economic and environmental considerations.

## 2. Materials and Methods

### 2.1. Starting Materials and Processing

Five compositions were studied, all with a 20 vol.% metallic binder phase. Two types of materials were studied: Ti(C,N)-based and WC-based. Regarding the metallic phase, compositions of Fe15Ni and Fe15Ni10Cr were prepared, with their nomenclature expressed in mass fractions (wt.%). The C content was controlled in the hardmetals to prevent either the formation of eta phase or graphite precipitation. Similarly, extra C was added in cermets to lower both their solidus and liquidus temperatures. The phase diagrams of these materials were compiled in a previous study [7]. These materials were compared with a commercial WC-Co hardmetal provided by CERATIZIT Luxembourg S. à r. l. The characteristics of the raw materials are listed in Table 1 and the theoretical composition of the processed materials is shown in Table 2.

The materials were processed via a powder metallurgy route. In the first step, the powders were milled in an attritor-type mill with both blades and vessel made of hardmetal. The programme performed was carried out with 10 mm hardmetal balls for 10 h in acetone. The powders were pressed into a uniaxial die at 200 MPa. The sintering followed the standard industrial sinter-HIP cycle performed at CERATIZIT facilities.

Two categories of samples were fabricated. Prismatic-shaped specimens measuring 7 × 7 × 20 mm^3^ were created specifically for conducting wear tests, while cylinders with a diameter of 3.3 mm and a length of 25 mm (ISO 3327—Piece type C [34]) were manufactured for transversal rupture strength (TRS) tests. The latter specimens, upon fracture, were subsequently utilised for oxidation tests.

### 2.2. Microstructural Characterisation

The samples were subjected to a metallographic preparation process through an automated polishing procedure involving grinding and polishing. Grinding was carried out using diamond grinding discs with particle sizes ranging from 120 μm down to 6 μm. For the final polishing stage, two cloths were employed that used diamond suspensions of 3 μm and 1 μm. Subsequently, Field-Emission Scanning Electron Microscopy (FE-SEM, FEI Teneo, Eindhoven, The Netherlands) was used to characterise their microstructures. Two different modes have been employed: BSE (Back-Scattered Electrons) and SE (Secondary Electrons). Furthermore, the quantification of the experimental metallic phase fraction and the average hard phase diameter was conducted through image analysis techniques and employing ImageJ software (version 1.54j). Their contiguity was calculated using Equation (1) [35]:

(1) $$C=\frac{D}{V_{Binder}^{n}}$$

where *D* = 0.2, *n* = 0.45, and *V_Binder_* is the volumetric fraction of the metallic binder, calculated experimentally.

On the other hand, the mean free path was calculated using Equation (2) [35]:

(2) $$\lambda =\frac{1}{1-C}\cdot \frac{V_{Binder}}{V_{Ceramic}}\cdot d_{Ceramic},$$

where *V_Binder_* and *V_Ceramic_* are the volume fraction of both phases, measured experimentally, and *d_Ceramic_* is the average hard phase diameter.

A minimum of 5 micrographs from distinct sample locations were used to calculate all the parameters mentioned above.

### 2.3. Hardness, Fracture Toughness, and TRS

Hardness was measured on the polished surface of the prismatic samples. This was achieved by performing a Vickers normalised test in 10 different regions, where a load of 30 kg was applied for 10 s (*HV*30). Fracture toughness (*K_Ic_*) was then determined using the Palmqvist method for hardmetals, employing the Shetty formula [36]. This involved measuring the cracks that were generated as a result of the hardness test and applying Equation (3) to calculate fracture toughness.

(3) $$k_{Ic}=0.15\sqrt{\frac{HV30}{\Sigma L}}$$

where *ΣL* is the summary of the length of the cracks.

The TRS of each material was determined by conducting a three-point bending test on 10 normalised cylinders (type C) following the ISO 3327:2009 standard [34]. The load speed employed during the test was set at 50 MPa/s.

### 2.4. Wear Tests

A ball-on-plate tribometer (UMT TriboLab, Bruker, MA, USA) was employed to conduct dry sliding wear tests. These tests involved reciprocal linear motion in an unlubricated environment at room temperature. Dry sliding experiments were conducted in accordance with the standard test procedure for linear reciprocating ball-on-flat sliding wear specified in the ASTM standard G133-05 [37], but employing a hardmetal ball (6 mm diameter, WC-6Co, 92.1 HRA) as the counter material. Various parameters such as load, frequency, and sliding time were varied to assess the wear characteristics of the materials under different conditions. Table 3 provides a comprehensive summary of all testing conditions. Each test was performed at least twice to ensure reproducibility.

The mass loss/gain of the samples was assessed with a precision of 10^−4^ g. Additionally, the coefficient of friction (COF) was measured during the tests. To determine the dimensions of the wear tracks, an optical profilometer (Zeta-20, KLA, Milpitas, CA, USA) was used to measure depth and width. At least 5 different regions of the wear tracks were analysed, excluding the tips. The measurement of surface roughness (R_a_) was conducted in arbitrary orientations before the experiments, and then after each test, but this time in perpendicular directions within the wear tracks. Furthermore, the wear tracks were examined using FE-SEM to identify the wear mechanisms present and determine the wear products.

To calculate the wear rate, a particular data analysis needs to be performed. The average area loss, ${\bar{A}}_{w}$ [mm^2^], is calculated from the width (*X*) and depth (*Y*) values from each 2D profile of the wear track using Equation (4) [38]:

(4) $${\bar{A}}_{w}=\sum_{i=0}^{n}0.5\left(Y_{i}+Y_{i-1}\right)\left(X_{i}-X_{i-1}\right)$$

Once the average wear area loss is calculated, the average depth, $\bar{D}$ [mm], is determined following Equation (5) [38]:

(5) $$\bar{D}=\frac{{\bar{A}}_{w}}{\bar{W}},$$

where $\bar{W}$ is the mean width of the wear track. On the other hand, the volume loss, V [mm^3^], can be calculated using Equation (6) [38]:

(6) $$V=\left[\frac{1}{3}\pi {\bar{D}}^{2}\left(3R-\bar{D}\right)\right]+{\bar{A}}_{w}\cdot l,$$

where R corresponds to the radius of the ball used as the counter material (3 mm) and l to the stroke length (5 mm). Finally, the wear rate, $W_{v}$ [mm^3^/mm], can be calculated considering V and the total sliding distance of the test, S [mm], using Equation (7) [38]:

(7) $$W_{v}=\frac{V}{S}$$

### 2.5. High-Temperature Oxidation Tests

**Figure 1:**
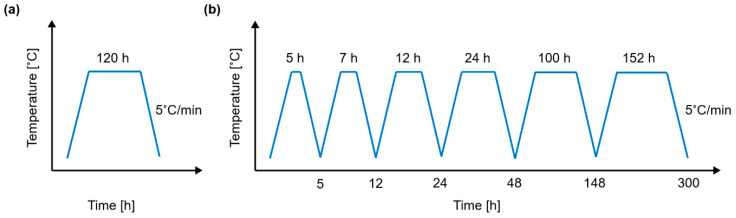
Scheme of the static-air high-temperature oxidation tests: (**a**) test for a fixed time of 120 h and (**b**) interval test for studying oxidation kinetics.

High-temperature oxidation tests were conducted in a muffle-type furnace under static air. Two different isothermal test conditions were employed (see Figure 1):

1. Exposing the samples to a constant temperature for a fixed time. The samples were heated at three different temperatures (500 °C, 800 °C, and 1000 °C) during a fixed time of 120 h.
2. Exposing the samples to a constant temperature for different periods. The samples were heated at 650 °C and 800 °C for up to 300 h. Measurements were taken at 5 h, 12 h, 24 h, 48 h, and 148 h. For this purpose, the samples were placed inside the furnace, heated until the test temperature was reached, maintained for the test period duration, cooled to room temperature within the furnace chamber, taken out for weight measurements (one specimen was kept out for FE-SEM characterisation), and then placed back inside the furnace and heated to the test temperature again.

The samples were carefully placed on a metallic mesh made from Nichrome wire with a diameter of 0.65 mm, ensuring the largest superficial area was exposed to the surrounding air. In all cases, two to five samples of each material were used to ensure measurement reliability and repeatability. As with the wear tests, the weights of the oxidation analyses have an accuracy of 10^−4^ g.

The oxidised surfaces were analysed by X-ray diffraction (XRD, Philips X’pert, Netherlands) using Cu kα (*λ* = 1.524 Å). The scanning was performed for 2θ angles between 20 and 80°, with steps of 0.04°, for 1 s. X’pert HighScore software 3.0 was employed to identify the phases formed in the material.

The thermal behaviour of the ceramic phase was studied via a Differential Thermal Analysis (DTA, Setaram SETSYS Evolution 16/18, Mougins, France). The analysis was conducted for Ti(C,N) and WC powders in an Al_2_O_3_ crucible under air flow (10 mL/min) up to 1200 °C with a heating rate of 20 °C/min.

## 3. Results and Discussion

### 3.1. Microstructure of Bulk Materials

**Figure 2:**
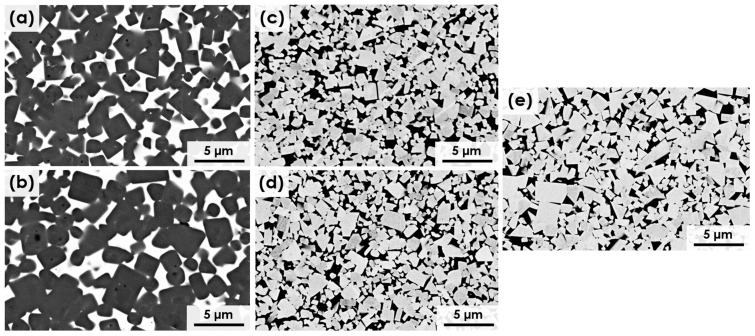
SEM micrographs (BSE mode) of the microstructure of sintered materials: (**a**) Ti(C,N)-FeNi, (**b**) Ti(C,N)-FeNiCr, (**c**) WC-FeNi, (**d**) WC-FeNiCr, and (**e**) WC-Co.

The microstructure of the five studied materials can be observed in Figure 2. The ceramic grains of all materials (the dark phase in Figure 2a,b and the bright phase in Figure 2c–e) are homogeneously distributed in the metallic matrix. In addition, all the materials present full relative density.

**Figure 3:**
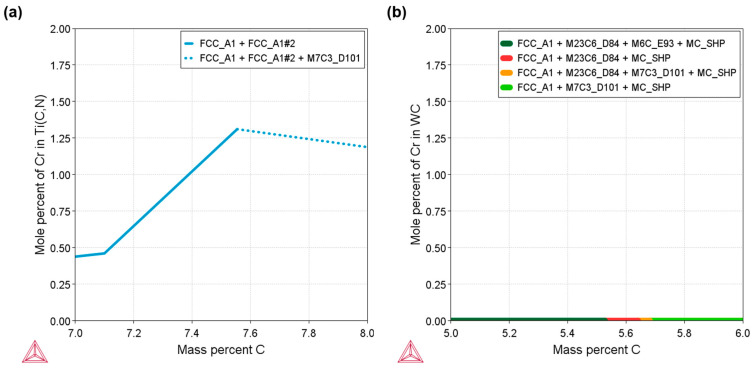
Thermodynamic calculation (using Thermo-Calc^®^–TCFE12 database) of Cr solubility in the main ceramic phases vs. the C content at 1000 °C in the studied systems: (**a**) solubility of Cr in the Ti(C,N) lattice (Theoretical C content of studied Ti(C,N)-FeNiCr—7.7 wt.% C) and (**b**) its solubility in the WC lattice (Theoretical C content of studied WC-FeNiCr—5.8 wt.% C).

In order to better understand the microstructure of the materials, some microstructural parameters were quantified and are shown in Table 4. WC-based materials present a smaller ceramic grain size than Ti(C,N)-based cermets. The addition of Cr into the Ti(C,N) metallic phase leads to larger size of ceramic grains compared to the Cr-free cermet. This might be influenced by the solubility of Cr in the Ti(C,N) FCC crystalline lattice, as illustrated in Figure 3a. In the case of WC-FeNiCr, the solubility of Cr in the WC crystalline lattice is null, as can be observed in Figure 3b. The carbide size of the Fe-based metal binders in the WC hard materials is slightly smaller than that of the Co-containing reference material. Roulon et al. [39] have previously reported this refining impact of Fe compared to Co in a WC-based hardmetal. In terms of contiguity, the materials based on WC exhibit higher values.

### 3.2. Mechanical Properties

Table 5 summarises the hardness, fracture toughness, and TRS results of the studied materials. The highest combination of values is observed for WC-FeNi, while WC-FeNiCr exhibits a slightly lower TRS value compared to the commercial cemented carbide, but this difference lies within the dispersion range. Additionally, WC-based materials (WC-FeNi, WC-FeNiCr, and WC-Co) display higher hardness and TRS values in comparison to the Ti(C,N)-based materials. These differences may be attributed to various features, including the difference in grain size and the chemical nature of the ceramic phase of these materials [40]. A correlation between TRS and both hardness and fracture toughness [41] is another factor influencing the higher TRS values of WC-based materials.

The addition of 10 wt.% Cr into the Fe15Ni metallic phase leads to a reduction in TRS values. A deeper analysis of the microstructure reveals the segregation of Cr-rich phases in the metal binder, with the presence of a Cr-rich phase surrounding the WC or Ti(C,N) ceramic phase due to the formation of M_7_C_3_-type carbides [31,32,42]. Part of the fracture of the material may be due to the propagation of cracks through this phase which is more brittle than the austenitic binder phase [43]. The effect of Cr on hardness and fracture toughness is less clear, as there are some variations in values but they are within the dispersion range.

**Figure 4:**
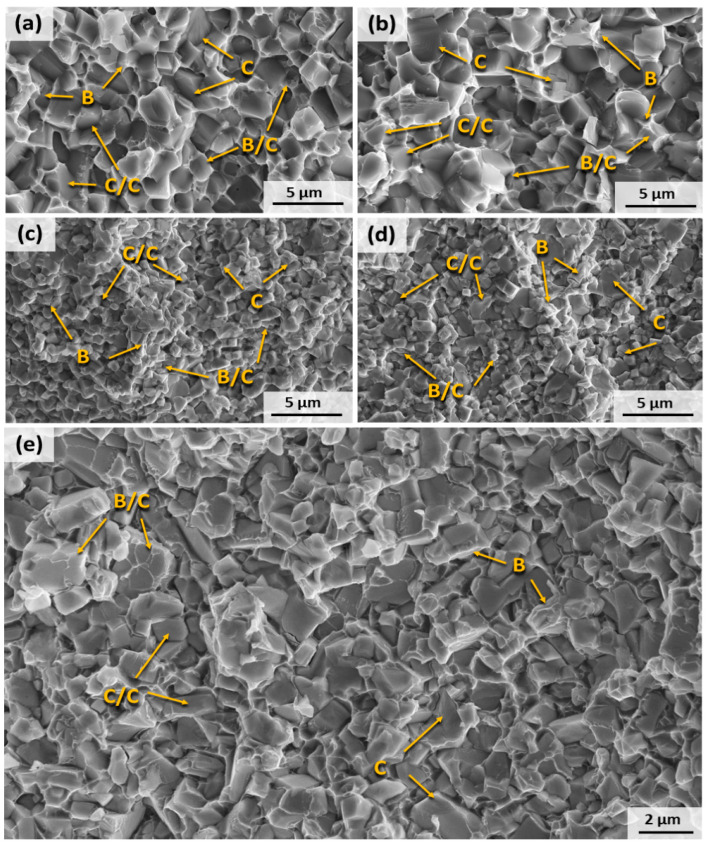
SEM micrographs (SE mode) of the fracture surface after TRS tests: (**a**) Ti(C,N)-FeNi, (**b**) Ti(C,N)-FeNiCr, (**c**) WC-FeNi, (**d**) WC-FeNiCr, and (**e**) WC-Co.

The fracture surface morphology resulting from the TRS tests of the studied materials is shown in Figure 4, where the different grain size between cermets and hardmetals (Table 4) is seen. The analysis of these images illustrates four distinct fracture modes: fracture along the interface between the ceramic phase and the binder (C/B), a ductile fracture of the binder phase (B), fracture between ceramic particles (C/C), and a transgranular fracture of a ceramic particle (C). The prevailing type of fracture observed in all materials was intergranular (C/B) fracture, with the material being toughened through a deflection mechanism. However, it is worth noting that cermets exhibit a higher occurrence of transgranular (C) fractures compared to WC-based materials. The propagation of cracks around the coarse grains of Ti(C,N) requires more energy, but they easily propagate through them, resulting in a (C) fracture [44]. The better metallic/ceramic interaction of the metallic binders with WC rather than Ti(C,N) [32] results in a higher amount of dimples in the WC interfaces and leads to type (B) fractures. This may explain the higher toughness and TRS of WC-based materials, as shown in Table 5.

### 3.3. Wear Tests

#### 3.3.1. Wear Response

The comparison of the surface roughness (Ra) before and after wear tests of each material can be found in Table 6. An increase in roughness is caused by the formation of a wear debris layer in the case of Ti(C,N)-based materials, whereas in the WC-based ones it may be due to the existence of material pull-outs and the formation of grooves. WC-FeNi shows a slightly lower increase in roughness compared to the other materials in milder conditions (setups 1, 2, and 3), whereas Ti(C,N)-FeNiCr shows the lowest increase in roughness in the more aggressive conditions (setups 3 and 5). Nevertheless, the variation in roughness is not very pronounced across all instances and falls within a similar range. 

**Figure 5:**
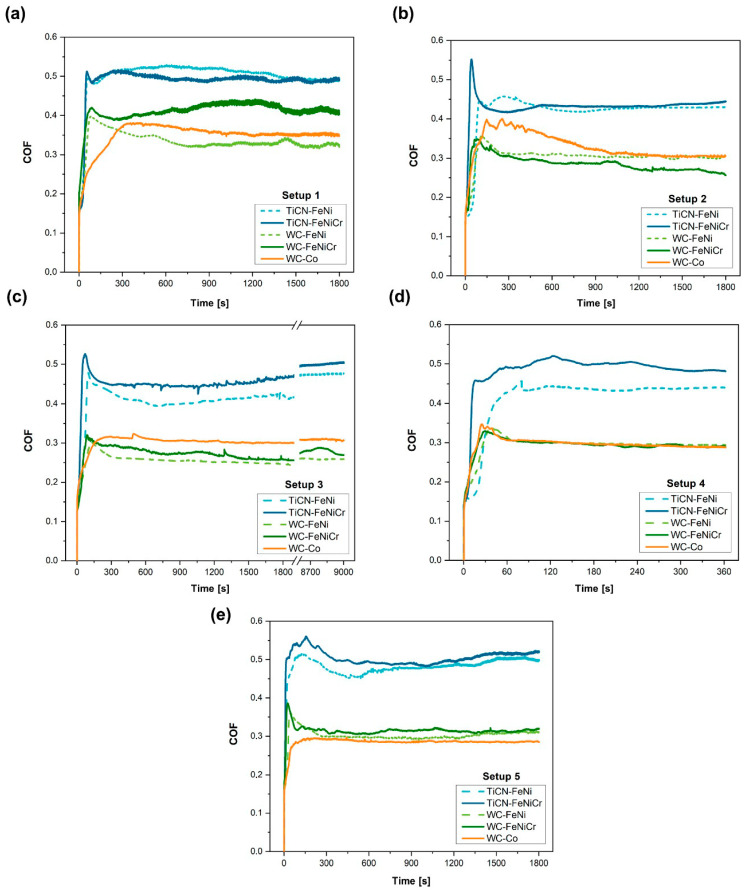
Evolution of the friction coefficient over the time for the five materials under (**a**) setup 1, (**b**) setup 2, (**c**) setup 3, (**d**) setup 4, and (**e**) setup 5.

To study the friction response of the samples under different wear conditions, the evolution of the COF throughout the tests was evaluated. The evolution of the COF vs. time under the different setups is plotted in Figure 5 and the steady-state COFs are listed in Table 7. During the first stage of sliding, the COF may vary and present non-definitive values due to the irregularities present on the surface. As the test continues, the wear track becomes uniform and even smoother and leads to a steady-state COF. Ti(C,N)-based materials exhibit a greater COF compared to WC-based materials in all conditions. The coefficient of friction exhibits a consistent decrease across all scenarios when the load is elevated from 10 to 30 N (setup 1 to 2). Conversely, when examining compositions involving the FeNi/FeNiCr binder, it becomes apparent that the COF experiences an upward trend as the test frequency rises (setup 3 to 5). This phenomenon can be attributed to the heightened frequency causing an escalation in fracture, which subsequently leads to the generation of rough debris, increasing friction [45].

**Figure 6:**
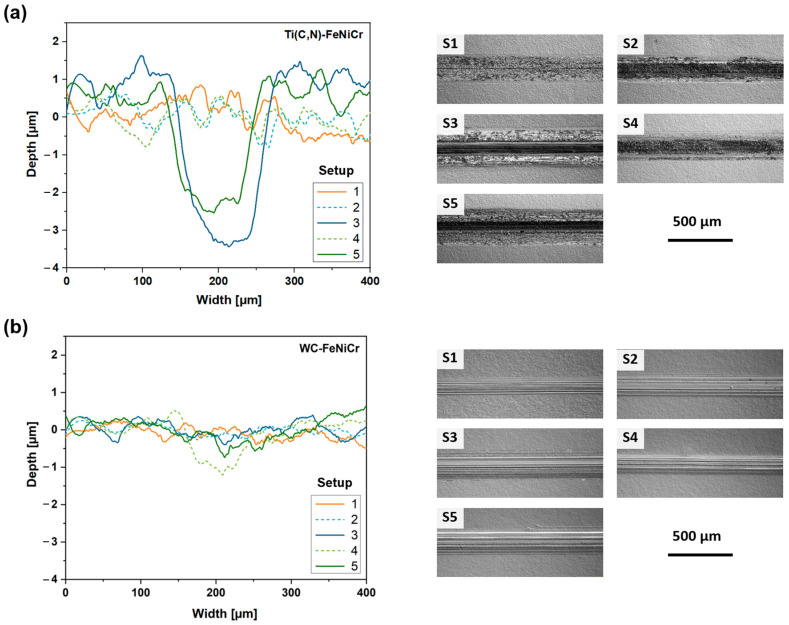
Wear profiles and wear tracks from each setup, measured/observed with optical profilometry, of (**a**) Ti(C,N)-FeNiCr and (**b**) WC-FeNiCr.

The mass variation does not follow a common pattern, showing fluctuations that correspond to losses/gains of mass but remain close to zero in all materials for all conditions. Since none of the five materials examined resulted in a substantial removal of material, the outcomes obtained are somewhat ambiguous. In the most extreme instances, any mass loss experienced is counterbalanced by the formation of oxides. Consequently, it becomes challenging to accurately determine the proportion of loss attributed to material removal versus the proportion of gain attributed to the presence of wear residues and oxides. Figure 6 presents the average wear profiles obtained from five different sections of the wear track for each condition applied to Ti(C,N)-FeNiCr and WC-FeNiCr. These two materials were chosen as they effectively demonstrate the behaviour exhibited by other studied Ti(C,N)-based and WC-based compositions. Notably, in Figure 6a, it can be observed that only setups 3 and 5 (which represent the most extreme conditions, at 90 m) exhibit a small maximum profile of 4 μm. Conversely, the WC-based materials do not display aggressive enough conditions to create a groove in the material. Instead, only marks on the substrate resulting from substrate abrasion are visible. Figure 6b provides a more comprehensive view of the wear tracks obtained through optical profilometry, allowing for a better understanding of the contrasting wear mechanisms seen between Ti(C,N)-based and WC-based materials. Further analysis of these mechanisms will be conducted in subsequent studies.

Table 8 shows the width of the wear tracks to better illustrate the physical impact on the materials. Variations exist among the materials formed by different ceramic phases, particularly in setup 3, where the wear conditions are more severe. However, overall, these disparities are minimal, with their values falling within a similar range, in several conditions. This observation is logical, considering that the penetration of the counter material is comparable (with minimal penetration in all cases), thereby limiting significant deviations in the track width.

In Table 9, the wear rate data of the studied materials are gathered. Notably, Ti(C,N)- and WC-based materials exhibit distinct behaviours. These outcomes are consistent with their previously observed wear characteristics. Ti(C,N)-based materials demonstrate a wider range of wear rate values under a test force of 30 N and a distance of 18 m (setups 2 and 4). Nevertheless, with an increase in the test distance to 90 m (setups 3 and 5), the difference in the wear rate between Ti(C,N)-FeNi and Ti(C,N)-FeNiCr diminishes. Therefore, it cannot be assumed that Cr has a clear effect on this behaviour. In addition, the difference in the sliding wear rate between Co-free WC-based hardmetals and commercial cemented carbide with a lower hardness is negligible. These results suggest that the composition of the metal phase has a minimum impact on wear behaviour compared to other mechanical properties. Instead, the ceramic phase has the most significant influence on this property. In this particular scenario, the decreased wear rate observed in WC-based materials could be attributed to either their elevated hardness [14], increased toughness [15], or reduced mean free path [46], aligning with other studies comparing cermets and hardmetals [14].

#### 3.3.2. Wear Mechanisms

**Figure 7:**
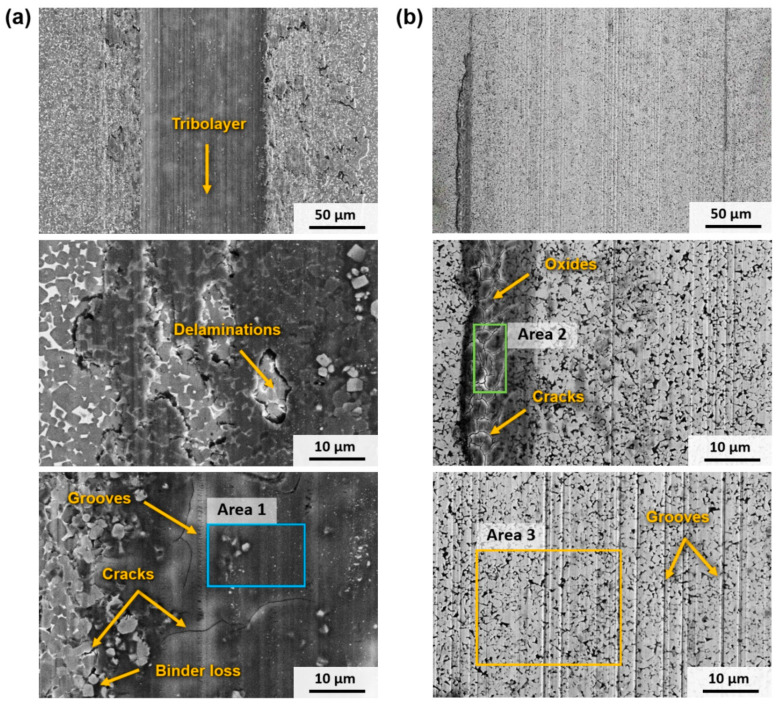
SEM micrographs (SE mode) of the wear track, under setup 5 conditions, of (**a**) Ti(C,N)-FeNiCr and (**b**) WC-FeNiCr.

In terms of wear mechanisms, the behaviour shown by Ti(C,N)-based and WC-based materials is different. In the case of Ti(C,N)-based materials, a layer of wear debris is formed in the central axis of the track and advances laterally as the test progresses under all setups. It was found that these residues first form in different areas of the track until, as the test continues, the residues coalesce, unifying into a single layer. This layer is mainly composed of Ti and Fe oxides, as can be seen in Table 10, which shows the composition of different areas of the wear tracks of Figure 7. The formation of TiO_2_ and Fe_2_O_3_ can be assumed following the analyses reported in previous studies [16,17]. The increase in the applied load promotes the formation of the tribolayer, as the temperature rises due to the increased frictional pressure [18]. This, in turn, facilitates the creation of oxides and the compaction of the tough debris, leading to the development of a compact and uninterrupted tribolayer on a portion of the worn surface. Consequently, the tribolayer acts as a protective barrier for the underlying bulk cermet. As a result, there is a noticeable reduction in the COF from setup 1 to the subsequent setups at higher loads, as indicated in Table 8. This finding aligns with previous studies [17] that have demonstrated a correlation between the lower shear strength of the tribolayer and a decrease in the COF. This layer of oxides does not cover the entire track but increases in width as the temperature in the contact zone increases, thus favouring the formation of oxides. This tribolayer also contains embedded fragments of ceramic particles and other wear debris from the counter material. Figure 7a shows that this is a brittle layer, as delamination and abrasion marks (ploughing and microcracking) can be found in this layer. 

**Figure 8:**
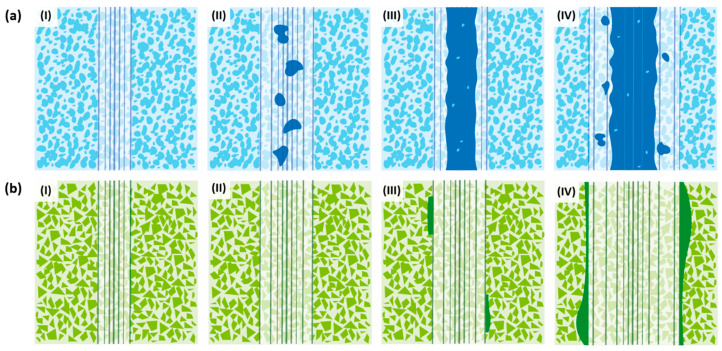
Wear evolution of hard materials: (**a**) Ti(C,N)-based cermets and (**b**) WC-based hardmetals.

Hence, as illustrated in Figure 8a, the wear mechanism of Ti(C,N)-based cermets under the conditions examined in this study can be described as (I) an initial abrasion of the contact surface; (II) oxide formation in the debris on the wear track caused by elevated temperature; (III) the fusion of the generated oxides with fragmented ceramic particles, leading to the creation of a continuous tribolayer along the central axis of the track; and (IV) the lateral expansion of the tribolayer as the experiment advances due to the emergence of new oxides, as well as abrasion and microcracking of the tribolayer due to the ongoing wear process.

In the case of materials based on WC, their behaviour differs. Their primary mode of wear is abrasion, which becomes more pronounced as the test conditions become more aggressive, resulting in the formation of larger grooves. The predominant form of mechanical wear was abrasion caused by plastic deformation rather than fracture. The absence of wear debris resulting from the fracture of WC particles or oxide asperities led to the occurrence of two-body abrasion, which differed from the cermets. Consequently, abrasive wear occurred through ploughing, as there was no indication of microcracking or wedge formation. According to Larsen-Basse [47], the initial stages of wear involve several processes such as polishing, abrasion, adhesion, the removal of the surface binder phase, and the extrusion of the binder phase from between WC grains. These processes are believed to contribute to carbide removal, carbide grain cracking, and particle fracture. Under the studied conditions, there are regions in the wear track that present higher deformation and losses of metallic binder between the WC grains. However, the ceramic particles were observed to remain well bonded to the metallic matrix during sliding and thus grain pull-out has not been noticed. Further studies conducted at higher loads and distances may characterise other types of wear. Furthermore, in tests conducted at 30 N and above, the formation of W, Fe, and Co oxides occurs, as indicated in Table 10, with the latter originating from the counter material. In addition, it can be observed that WC-based materials experience more contamination from the counter material, particularly with a higher presence of Co. This could be attributed to the fact that WC-based hard materials possess higher hardness, leading to increased wear between the counter ball and the material, resulting in the presence of these residues that adhere to the wear track. Hence, the wear process of WC-based cemented carbides—in the specific experimental conditions outlined in this research—is illustrated in Figure 8b and involves the following phases: (I) the initial development of abrasion marks on the surface of the material; (II) the widening of the worn track through the creation of additional abrasion marks via ploughing; (III) the generation of oxides on the track edges primarily caused by contamination from the counter- ball; and (IV) the progression of the worn track through abrasion, erosion of the metal binder, and the production of new wear debris at the boundaries of the track.

### 3.4. High-Temperature Oxidation

#### 3.4.1. Oxidation Behaviour

**Figure 9:**
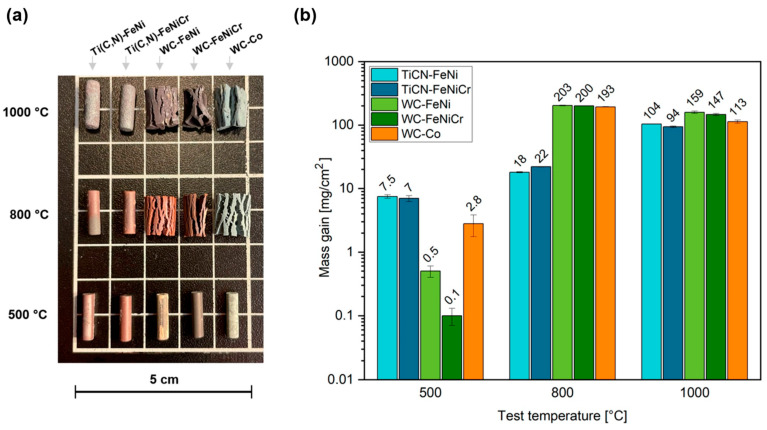
The results of the oxidation tests performed at set temperatures for 120 h: (**a**) appearance of the samples after oxidation and (**b**) mass gain of the materials during the tests.

No significant alterations in the morphology of any of the samples oxidised at 500 °C were observed, but a change in the surface colour occurred, as illustrated in Figure 9a. However, as the temperature is raised, a catastrophic oxidation of the WC-based materials occurs, resulting in a considerable expansion and corresponding cracking of the cylindrical specimens. This expansion caused the specimen to lose its original shape when the experiment was conducted at 800 or 1000 °C.

In terms of mass gain (Figure 9b), the values at 500 °C are relatively low for all materials. Comparatively, Ti(C,N)-based materials exhibit higher mass gain values when compared to WC-based materials. Notably, at this temperature, WC-Fe-based materials demonstrate lower mass gain than the commercial-grade material.

At 800 °C, both Ti(C,N)-FeNi and Ti(C,N)-FeNiCr exhibited comparable mass gains, which were roughly ten times lower than that of their WC-based respective materials. The WC-Co showed a similar value to the alternative binders. When exposed to temperatures of 1000 °C, materials based on Ti(C,N) exhibited an increase in mass, yielding similar values. Conversely, the WC-based materials displayed a lower increase in mass during the 800 °C test, which can be attributed to the volatilisation of oxides. It becomes evident that the impact of the metal phase on oxidation behaviour is only significant under test conditions that do not lead to oxidation of the ceramic phase, such as in the case of WC at 500 °C. At higher temperatures, the oxidation of the ceramic phase becomes more prominent, making it challenging to discuss the influence of the metallic binder on mass gain.

**Figure 10:**
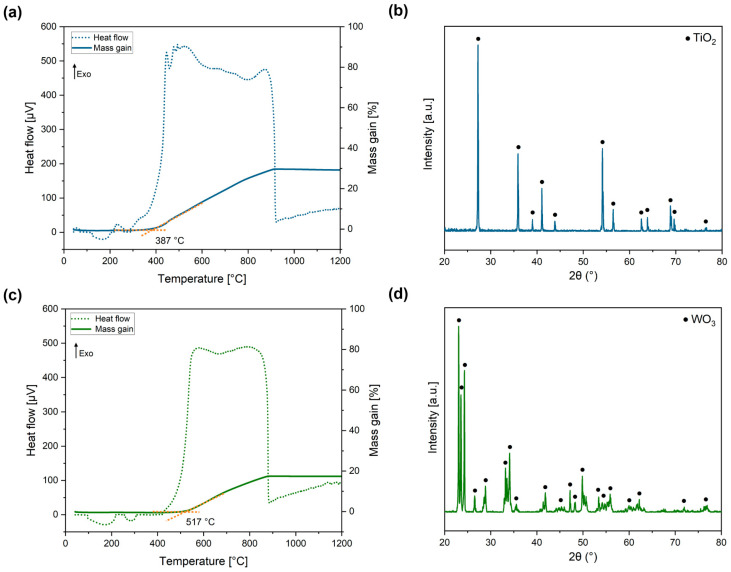
DTA-TG curves of the oxidation of the ceramic powders (left) and XRD patterns (right) of the oxidation products of (**a**,**b**) Ti(C,N) and (**c**,**d**) WC.

A DTA-TG analysis was performed on Ti(C,N) and WC powders to determine the temperature at which their oxidation starts. The results shown in Figure 10a,c indicate that both materials exhibit exothermic peaks and a subsequent increase in mass attributed to oxidation phenomena.

The DTA-TG curve of the Ti(C,N) powders (Figure 10a) displays an endothermic peak at 387 °C, which indicates the start of the oxidation reaction. In the case of WC powders, their oxidation reaction occurs at a higher temperature, specifically 517 °C. The resulting oxides from the DTA test (Figure 10b,d) are identified as TiO_2_ and WO_3_ for the respective ceramic powders. The total mass gain percentage of WC reaches 17.4%, which is consistent with previous studies and also supports the dependence of the beginning of the oxidation reaction on the particle size of WC [48]. 

#### 3.4.2. Oxidation Kinetics

**Figure 11:**
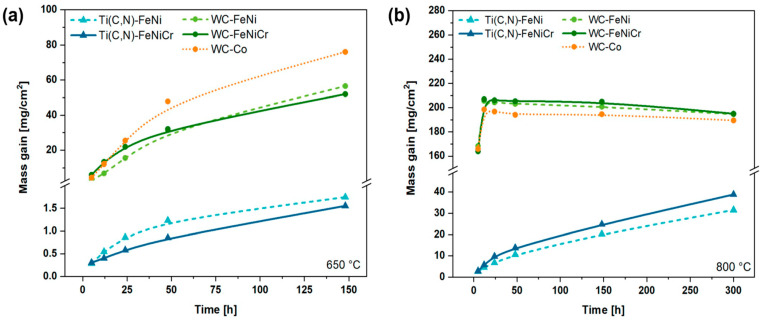
Experimental curves of the oxidation kinetics of the studied materials at a temperatures of (**a**) 650 °C and (**b**) 800 °C.

High-temperature oxidation behaviour was also evaluated by studying oxidation kinetics, as shown in Figure 11. At 650 °C (Figure 11a), it is evident that all five materials exhibit an increasing trend in their kinetics. Notably, the cermets display a mass gain of several mg/cm^2^, whereas the WC-based hardmetals exhibit a higher mass gain. Generally, the presence or absence of Cr in these compositions has a negligible impact, except for WC-Co, which shows a higher mass gain compared to WC-FeNi and WC-FeNiCr. This can be attributed to the oxidation products resulting from Co, which aligns with the lower-temperature oxidation outcomes at 500 °C illustrated in Figure 9b. In the case of the 800 °C tests (Figure 11b), the behaviour of the cermets remains similar to that observed in the 650 °C tests. However, the WC-based hardmetals experience a volatilisation of their oxides, leading to a loss of mass over time. This indicates a rapid oxidation of WC and a subsequent loss of oxides through oxide volatilisation.

To understand this oxidation from another point of view, Equation (8) (taken from [23]) was employed to parameterise the oxidation kinetics of the materials.

(8) $$\left(\frac{\Delta m}{S}\right)=k_{n}\cdot t^{n}+C$$

where ∆*m* denotes the mass gain, *s* represents the materials’ surface exposed to air, *t* is the oxidation time, *k_n_* denotes the oxidation rate constant, *n* represents the oxidation exponent, and *C* is a constant that marks the oxidation that occurred before the isothermal condition was obtained. A coefficient of 0.5 would represent an ideal parabolic response, indicating a protective growth rate. However, deviations in the exponent value arise from diffusion phenomena and the occurrence of cracks in the oxide layer, leading to mixed behaviour. The values of *k_n_* and *n*, which were derived through the process of fitting using Equation (8), are presented in Table 11. Additionally, the coefficient of determination (R^2^) is included as a measure of the adequacy of the fitting.

It can be seen that the values of *n* from Ti(C,N)-based cermets at both 650 and 800 °C are within a range of 0.4 to 0.6; values that correspond to quasi-parabolic behaviour and whose deviation from the ideal value is due to the composite nature of the materials. Furthermore, there are fast diffusion paths in localised areas that influence the oxidation process, which also affects this behaviour, as reported in other studies [23,49]. However, quasi-parabolic oxidation kinetics are associated with the existence of a protective layer. The diffusion of ions (O_2_^−^ and Fe^3+^) occurs in both inward and outward directions through the protective TiO_2_ layer in this system, resulting in a parabolic pattern. Nevertheless, the presence of cracks, gaps, or other imperfections in the oxide layer introduces variations in the behaviour of O_2_^−^, causing it to react with the underlying material. Consequently, the oxidation process in this system can be characterised as a combination of both diffusion and direct reactions, as previously stated. On the other hand, at 650 °C, the oxidation rate is lower in the Cr-containing cermet, although the difference is minimal. However, when increasing the temperature to 800 °C, the values increase, leading to Ti(C,N)-FeNiCr having a higher oxidation rate.

Regarding the oxidation kinetics of hardmetals, at 650 °C, their exponential coefficients exhibit greater variation, which raises doubts about the materials’ parabolic nature. Instead, they demonstrate purely exponential behaviour without any protective characteristics. The commercial-grade WC-Co demonstrates the highest oxidation rate of the studied materials. Conversely, when considering alternative hardmetals, the Cr addition appears to diminish the oxidation rate. On the other hand, at 800 °C, the materials do not conform to an exponential curve but rather follow a straight line (n = 1) with a decreasing slope. This decline in slope is attributed to the loss of mass caused by the volatilisation of WO_3_, as it starts to volatilise at a temperature of 750 °C, with a significant increase in the volatilisation rate observed once the temperature exceeds 900 °C [50].

#### 3.4.3. Scale and Transitions of Oxides: Oxidised Surface and Cross-Section Morphologies

**Figure 12:**
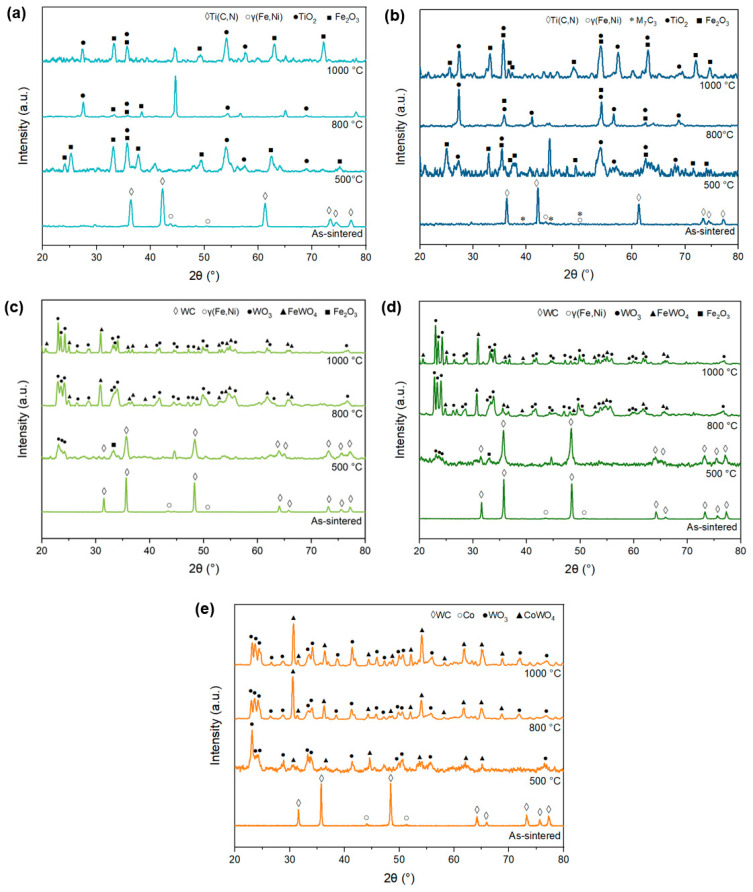
XRD patterns of the surface products, after oxidation tests at different temperatures for 120 h, of (**a**) Ti(C,N)-FeNi, (**b**) Ti(C,N)-FeNiCr, (**c**) WC-FeNi, (**d**) WC-FeNiCr, and (**e**) WC-Co.

Superficial oxidation products were identified through the XRD analysis of the samples. The XRD patterns presented in Figure 12a,b represent the Ti(C,N)-based cermets with FeNi and FeNiCr binders, respectively. Both polished sintered materials consist of Ti(C,N) and austenite. Following the oxidation tests, new superficial phases were detected, specifically Ti and Fe oxides, including TiO_2_ and Fe_2_O_3_. It is important to note that these analyses were conducted on the surface of the samples, which introduced experimental noise due to surface curvature and roughness. 

The XRD patterns of the WC-based materials with FeNi and FeNiCr binders are shown in Figure 12c,d. The sintered materials before the oxidation tests consisted of WC and austenite. The analysis of the peaks detected after the 500 °C test revealed that they still corresponded to WC, indicating that only a slight amount of oxidation had occurred. This finding is consistent with the results of the mass gain measurements shown in Figure 9. These results demonstrate that the higher mass gain of cermets during the test at 500 °C is due to the formation of TiO_2_ and that the oxidation reaction of WC in hardmetals has not yet reached a stable level (as shown in Figure 10c). However, peaks corresponding to WO_3_ and Fe_2_O_3_ were also observed. The peaks detected during the tests conducted at higher temperatures (800 °C and 1000 °C) were similar and corresponded to mixed WO_3_ and FeWO_4_. The findings from the analysis of the commercial-grade WC-Co (Figure 12e) align with previous research conducted on this composition [26,51], confirming the formation of WO_3_ and CoWO_4_. The behaviour observed at temperatures of 800 °C and 1000 °C exhibits similarities, while, at 500 °C, as in the case of WC-FeNi and WC-FeNiCr, WC does not undergo complete oxidation. Nevertheless, in this instance, the detection of WC peaks is absent, likely attributed to the larger size of the oxide layer.

**Figure 13:**
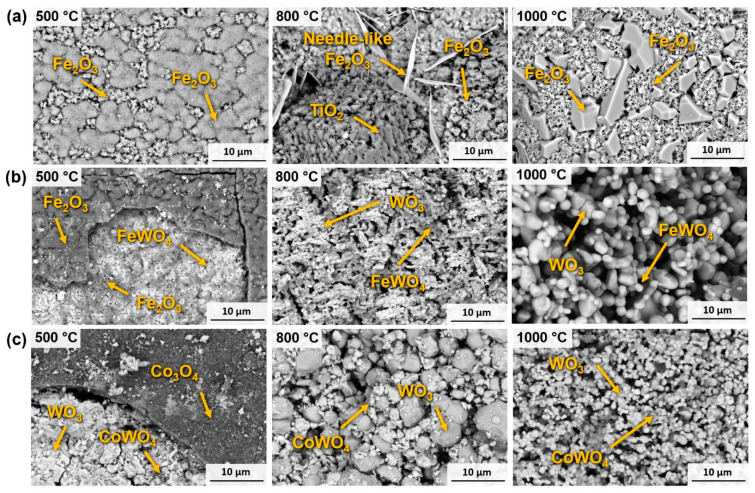
The surfaces of oxidised samples, characterised by SEM (SE mode), of (**a**) Ti(C,N)-FeNiCr, (**b**) WC-FeNiCr, and (**c**) WC-Co.

SEM was employed to analyse the surface oxides to demonstrate the results obtained by XRD. In the case of Ti(C,N)-FeNiCr at 500 °C (in Figure 13a), the outer layer consists of Fe_2_O_3_, exhibiting two distinct geometries: one compact and the other needle-like. At 800 °C, the surface oxides exhibit better differentiation between the two types of Fe_2_O_3_ geometries. Additionally, the presence of TiO_2_ becomes noticeable at this temperature. When the test temperature reaches 1000 °C, the outer layer is once again exclusively composed of Fe_2_O_3_. However, the morphology of this oxide presents to two variants at this temperature: one porous and the other forming a solid geometry. All these results are in accordance with the XRD measurements shown in Figure 12b.

In the case of WC-FeNiCr, there is a noticeable difference in behaviour. When subjected to testing at 500 °C, a brittle outer layer composed of Fe_2_O_3_ was observed. Figure 13b illustrates that this layer had fractured, revealing the oxides that developed beneath it. These oxides correspond to Fe_2_O_3_, which contributes to the growth of the upper layer, as well as FeWO_4_. The DTA-TG test indicates that the WC had not yet reached its exothermic peak at this temperature. However, it was sufficient to oxidise a small portion, resulting in the formation of a mixed oxide consisting of W and Fe in the intermediate layer adjacent to the substrate. At 800 °C and 1000 °C, it is noteworthy that the size of the oxides may differ, but their composition remains similar, comprising WO_3_ and FeWO_4_. At these elevated temperatures, there is no longer any evidence of Fe_2_O_3_, as it combines with WO_3_ to form a mixed oxide in accordance with XRD measurements from Figure 12d and with previous studies on WC-Fe-based hardmetals [28].

Regarding WC-Co, the behaviour it displayed is quite similar to that of other WC-based materials. At 500 °C, oxidation products were formed as a delicate outer layer composed of Co_3_O_4_ which had been fractured; see Figure 13c. This fracture allows for the observation of the oxides that have formed in the underlying layers, specifically WO_3_ and CoWO_4_. As the temperature increased further, the oxide became entirely porous and brittle, consisting of loosely bonded oxide particles. The oxides identified at both temperatures are WO_3_ and CoWO_4_.

**Figure 14:**
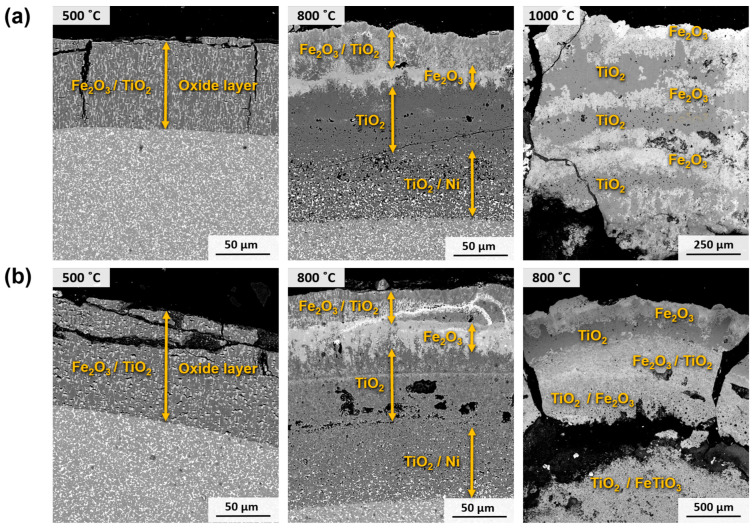
SEM micrographs (BSE mode) of the cross-section of samples after oxidation for 120 h at different temperatures in air: (**a**) Ti(C,N)-FeNi and (**b**) Ti(C,N)-FeNiCr.

A further analysis of the oxides and their formation within the material is achieved by examining the cross-section of the tested specimens, as depicted in Figure 14. At a temperature of 500 °C, the cermets exhibit a blended layer consisting of TiO_2_ and Fe_2_O_3_. Notably, the Fe_2_O_3_ exists in the form of domains encircled by a continuous TiO_2_ matrix. At 800 °C, these oxidation products are formed in different layers that are comparable for both materials. These comprise an outer layer composed of Fe_2_O_3_ and TiO_2_, an intermediate layer consisting of Fe_2_O_3_, and an inner layer exclusively formed of TiO_2_. Within these layers, small regions of the metallic phase, enriched in Ni due to its superior resistance against oxidation, can be observed. Additionally, Fe and Cr have diffused to react with O_2_, resulting in the formation of oxides in the outermost layers. Upon reaching a temperature of 1000 °C, a collapse occurs in the oxide layers, facilitating the more effortless penetration of O_2_. Coupled with the heightened diffusivity of O_2_ due to the elevated temperature, the entire material transforms into oxides, with no presence of the cermet substrate. In this scenario, the segregation and arrangement of the mixed oxides become more pronounced. Consequently, the outer layer comprises Fe_2_O_3_, followed by a layer of Fe_2_O_3_ and TiO_2_. The inner region of the material consists of a combination of TiO_2_ and the complex oxide FeTiO_3_.

**Figure 15:**
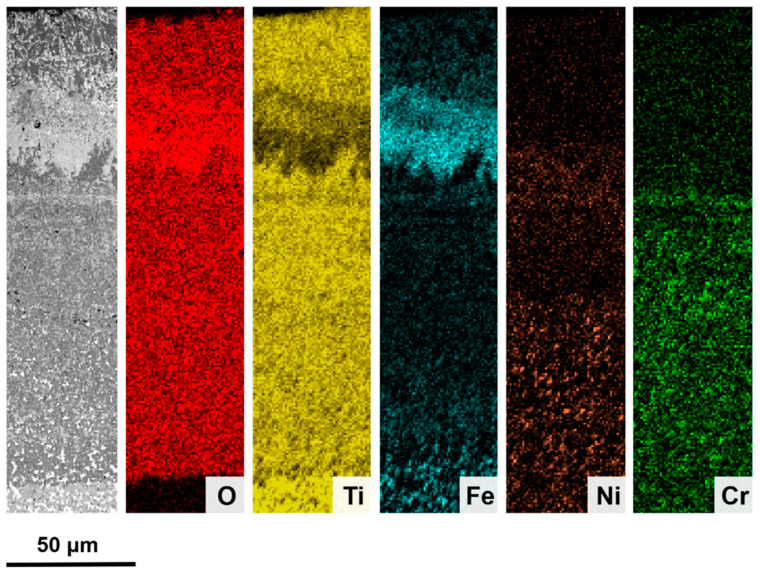
EDS map of the cross-section of Ti(C,N)-FeNiCr after oxidation for 120 h at 800 °C in air.

Figure 15 shows an EDS map depicting the distribution of elements in Ti(C,N)-FeNiCr after the oxidation test at 800 °C for 120 h. The map reveals that the outermost layer exhibits a blend of Fe and Ti oxides. The next oxide layer consists mainly of an almost pure Fe oxide layer. This layer marks the diffusion limit of the Ni^2+^ and Cr^3+^ ions that are within the other Ti oxides in the inner layer. Additionally, the map demonstrates that the metallic regions present in the inner TiO_2_ layer exhibit a composition gradient. As previously mentioned, the metallic regions closer to the surface exhibit a higher concentration of Ni, whereas, towards the substrate, the metal’s composition gradually approaches its theoretical composition.

**Figure 16:**
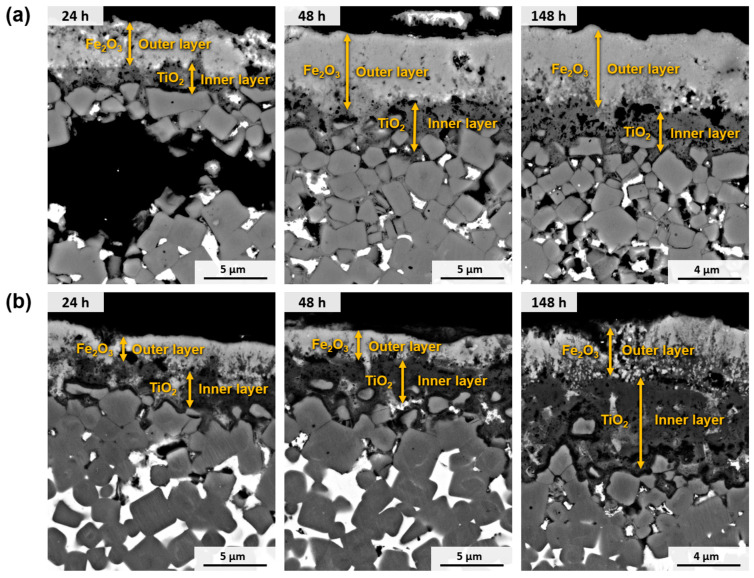
SEM micrographs (BSE mode) of the cross-section of samples after oxidation for different periods at 650 °C in air: (**a**) Ti(C,N)-FeNi and (**b**) Ti(C, N)-FeNiCr.

During experiments conducted at a temperature of 650 °C for different periods, a noticeable disparity in the behaviour of materials resulted from the addition of Cr, as shown in Figure 16. Both materials exhibited a biphasic oxide layer consisting of an external layer of Fe_2_O_3_ and an internal layer of TiO_2_. However, in the case of Ti(C,N)-FeNi, the outer Fe_2_O_3_ layer was found to be thicker. This discrepancy can be attributed to the presence of Cr in the metallic phase, as reported in other studies [25]. Consequently, the material containing Cr exhibited a lower increase in mass, as depicted in Figure 9b. In addition, in the case of Ti(C,N)-FeNiCr, there were regions of TiO_2_ within the Fe_2_O_3_ layer. This occurrence can be attributed to the fact that Cr, which does not form single oxides, influences the solubilities and distribution of the Fe and Ti oxides.

**Figure 17:**
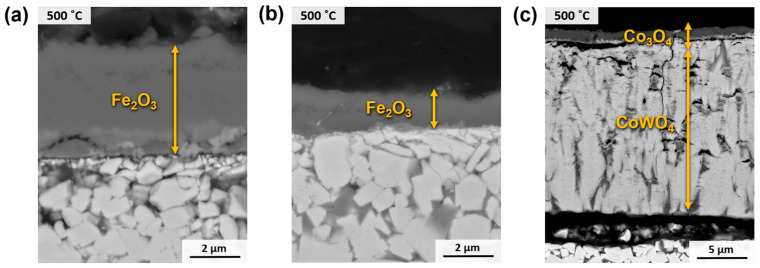
SEM micrographs (BSE mode) of the cross-section of samples after oxidation for 120 h at 500 °C in air: (**a**) WC-FeNi, (**b**) WC-FeNiCr, and (**c**) WC-Co.

As previously discussed, the oxidation mechanisms of hardmetals differ significantly from those of cermets. The results of the 120 h oxidation tests indicate that only the samples exposed to 500 °C exhibited an oxide–substrate layer configuration. However, during the 800 °C and 1000 °C tests, the metal–ceramic composite underwent complete oxidation, resulting in a brittle oxide configuration. The results of the 500 °C test are presented in Figure 17. In the case of WC-FeNi and WC-FeNiCr, a layer of Fe_2_O_3_ a few microns thick can be seen, as previously confirmed by the observation of surface oxides. For WC-Co (Figure 17c), two oxide layers are visible: an outer layer corresponding to Co_3_O_4_ and a larger mixed layer of WO_3_ and CoWO_4_, which is responsible for the higher mass gain shown in Figure 9b.

**Figure 18:**
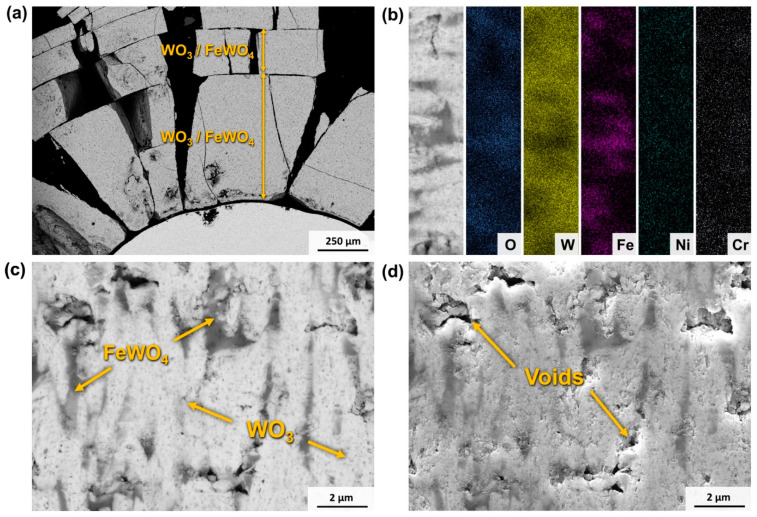
SEM micrographs of the cross-section of WC-FeNiCr after a 650 °C test for 48 h: (**a**) low magnification (BSE mode), (**b**) EDS mapping of a high-magnification oxide region, (**c**) high magnification (BSE mode), and (**d**) high magnification (SE mode).

The oxides formed on the hardmetals during the 800 °C and 1000 °C tests were too brittle to be mounted on resin for a cross-sectional analysis. Therefore, the cross-section of WC-FeNiCr exposed to air at 650 °C for 48 h is presented in Figure 18 to show their oxide characteristics, as the XRD showed that the composition of the oxides does not vary. The material exhibits a single oxide layer with a width of more than 1 mm that grows radially, leading to its fracture due to swelling, as can be seen in Figure 18a. The layer is composed of a FeWO_4_ matrix with WO_3_ domains within it. As shown in Figure 18b, Ni and Cr are distributed in both phases. The abnormal oxidation of these materials may be due to the formation of a porous structure of in the oxide layer. Figure 18d illustrates the significant presence of pores within the layer, providing pathways for O^2−^ ions to permeate through and further oxidise the substrate.

#### 3.4.4. Oxidation Mechanisms

The oxidation behaviour of the materials can be divided into two groups due to the different chemical natures of their ceramic phase. Thus, the oxidation behaviour exhibited by hardmetals and cermets is different. Based on the results shown above, several oxidation mechanisms are proposed for these materials.

**Figure 19:**
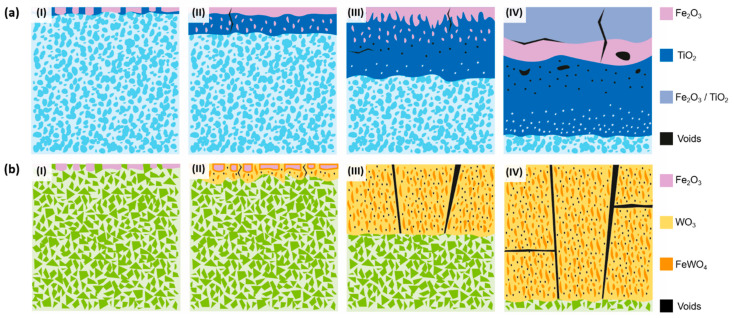
Oxidation evolution of hard materials: (**a**) Ti(C,N)-based cermets and (**b**) alternative WC-based hardmetals.

The oxidation of Ti(C,N)-based cermets (Figure 19a) can be described as follows:

- The oxidation process of Ti(C,N)-based cermets starts with the simultaneous oxidation of both their metallic and ceramic phases through direct contact with atmospheric O_2_. Consequently, the metallic and ceramic phases undergo oxidation through the formation of Fe_2_O_3_ and TiO_2_, respectively.
- Resulting from the inward diffusion of Fe^3+^, a Fe_2_O_3_ layer is generated on the outer surface of the material. However, it remains permeable to O^2−^, allowing the oxidation of TiO_2_ to continue within the material.
- As the internal TiO_2_ layer becomes denser, the inward diffusion of O^2−^ is controlled, and the primary mode of oxidation shifts to the outward diffusion of the metal ions. This phenomenon aligns with Wagner’s theory, which suggests that the oxidation rate is governed by the diffusion of ions. This correlation is further supported by the observation that the oxidation kinetics conform to a parabolic function, as previously demonstrated [52]. This reaction, involving Ti(C,N), produces the oxidation product and generates CO, CO_2_, and N_2_, which contribute to the defects and voids formed in the oxide layer.
- Over time, the external layer displays a combination of Ti and Fe oxides. Within an intermediate layer, a Fe_2_O_3_ region emerges, indicating the diffusion limit of Ni^2+^ and Cr^3+^, which are dispersed in the innermost regions of the material, contributing to other Ti and Fe oxides. The innermost region, consisting primarily of TiO_2_, continues to expand with time. As we near the metallic substrate, Ni-rich metallic regions are present within this oxide layer, given its lower reactivity compared to Fe and Cr. Fe^3+^ diffuses more rapidly towards the surface, while Cr^3+^ integrates into adjacent oxides, potentially forming a stoichiometric composition of Cr_x_Fe_2−x_TiO_5_.

WC-based hardmetals exhibit a different oxidation evolution, as shown in (Figure 19b). Their oxidation process can be divided into several stages:

- Initially, there is a selective oxidation of the metallic phase, resulting in the formation of Fe_2_O_3_ on the surface.
- Subsequently, the WC component begins to oxidise, leading to void formation and the growth of WO_3_ in the radial direction. As a consequence, a reaction occurs between WO_3_ and the Fe_2_O_3_ formed during the oxidation of the metallic phase, resulting in the formation of FeWO_4_ domains within the WO_3_ oxide.
- During the oxidation of WC, the production of CO_x_ leads to a pressure difference within the oxide. This pressure difference induces the formation of cracks, which in turn promote the growth of the oxide. These cracks serve as channels through which O_2_ can directly react with the substrate material. Furthermore, the porous nature of the oxide facilitates the inward diffusion of O^2−^, thereby enhancing the overall oxidation rate.
- The oxide continues to grow, leading to catastrophic oxidation and the collapse of the material.

## 4. Conclusions

The current study investigates the performance of cermet and hardmetal compositions based on Ti(C,N) and WC with Co-free binders under high-temperature oxidation and wear conditions. Furthermore, the effect of a Cr addition on these properties is examined, and these alternative compositions are compared to commercial-grade WC-Co. Based on the obtained results, the following conclusions may be drawn:

- Regarding the mechanical properties of the hard materials, WC-based materials exhibit superior hardness, toughness, and TRS compared to cermets. Specifically, WC-FeNi demonstrates higher levels of hardness and TRS when compared to the commercial-grade material (an increase of 7% and 9%, respectively). The addition of Cr results in a reduction in TRS (13% for the cermet and 17% for the hardmetal), likely attributed to the precipitation of M_7_C_3_-type carbides surrounding the Ti(C,N) and WC particles. Nevertheless, the TRS values of WC-FeNiCr are in the range of those found for WC-Co. The impact of Cr on hardness and fracture toughness is less clear.
- The results of the wear tests indicate that WC-based hardmetals exhibit a lower COF against a cemented carbide ball, as well as a lower wear rate under all test conditions. For instance, under the most aggressive conditions—setup 5—the cermets exhibit a COF of approximately 0.5, while hardmetals have a COF of about 0.3. Additionally, the wear rate of cermets is six times greater than that of hardmetals. Increasing the contact load resulted in a higher wear volume and a marginal reduction in the COF. The primary disparity in wear behaviour can be attributed to the distinct characteristics of the materials’ ceramic phase, as the metallic binder does not appear to have a significant impact on the wear process. In the case of Ti(C,N)-based cermets, the wear mechanism is attributed to the formation of a tribolayer consisting of Ti and Fe oxides, as well as wear debris. Conversely, the wear mechanism in WC-based hardmetals primarily involves abrasion through ploughing.
- Ti(C,N)-based materials exhibit favourable high-temperature oxidation behaviour by forming a protective layer composed of TiO_2_. This characteristic is due to the fact that the oxidation kinetics of cermets can be adjusted to an exponential law that closely resembles parabolic behaviour but is influenced by various factors. This behaviour provides a significant advantage over the volatile oxides of W. Thus, cermets present a much lower mass gain compared to WC-based hardmetals, which experience catastrophic oxidation at high temperatures (e.g., at 800 °C for 120 h, cermets experience a 10-times lower mass gain than their respective alternative hardmetals). In addition, the addition of 10 wt.% of Cr to the FeNi binder does not seem to be enough for the formation of a protective layer in any scenario. Nevertheless, it does influence the solubilities and distribution of oxides.

## Figures and Tables

**Table 1:** Characteristics of the initial raw powders.

Raw Powders	Provider	Characteristics
Ti(C_0.5_,N_0.5_)	Treibacher Industrie AG (Althofen, Austria)	FSSS = 1.66 μm
WC	–	–
Fe	–	FSSS = 2.32 μm
Ni	Vale International SA (Saint-Prex, Switzerland)	FSSS = 2.25 μm
Cr	SkySpring Nanomaterials (Houston, TX, USA)	D_50_ = 5 μm
C	Thermax^®^, Cancarb (Medicine Hat, AB, Canada)	FSSS = 0.40 μm

**Table 2:** Composition of studied materials.

Material	Ceramic Phase 80 vol.%		Metallic Phase 20 vol.%	
	Type	[wt.%]	Type	[wt.%]
Ti(C,N)-FeNi	Ti(C,N)	71.77	Fe15Ni	28.23
Ti(C,N)-FeNiCr	Ti(C,N)	71.97	Fe15Ni10Cr	28.03
WC-FeNi	WC	88.62	Fe15Ni	11.38
WC-FeNiCr	WC	88.71	Fe15Ni10Cr	11.29
WC-Co	WC	87.54	Co	12.46

**Table 3:** Summary of the wear conditions employed in each setup for the five materials.

Setup	Counter Material	Load [N]	Frequency [Hz]	Time [s]	Stroke Length [mm]	Sliding Distance [m]
1	WC-Co 6 mm ⌀ Ball	10	1	1800	5	18
2		30	1	1800	5	18
3		30	1	9000	5	90
4		30	5	360	5	18
5		30	5	1800	5	90

**Table 4:** Microstructural characteristics measured by image analysis.

Material	Ceramic Diameter [µm]	Metallic Phase [%]	Free Mean Path [µm]	Contiguity
Ti(C,N)-FeNi	1.60 ± 0.66	20.5 ± 0.3	0.82 ± 0.13	0.38
Ti(C,N)-FeNiCr	1.92 ± 0.79	20.4 ± 0.6	0.92 ± 0.26	0.39
WC-FeNi	0.94 ± 0.45	20.2 ± 0.5	0.39 ± 0.05	0.42
WC-FeNiCr	0.87 ± 0.44	19.7 ± 0.4	0.36 ± 0.01	0.42
WC-Co	1.00 ± 0.50	19.8 ± 0.2	0.41 ± 0.05	0.42

**Table 5:** Vickers hardness, fracture toughness, and transversal rupture strength of the studied materials.

Material	*HV*30 [kg/mm^2^]	*K_IC_* [MPa·m^1/2^]	TRS [N/mm^2^]
Ti(C,N)-FeNi	1275 ± 36	12.2 ± 1.5	2210 ± 132
Ti(C,N)-FeNiCr	1246 ± 56	11.4 ± 1.5	1915 ± 47
WC-FeNi	1400 ± 22	13.5 ± 0.2	3533 ± 101
WC-FeNiCr	1458 ± 25	13.7 ± 0.5	2924 ± 145
WC-Co	1298 ± 14	15.3 ± 0.7	3198 ± 122

**Table 6:** Initial surface roughness and increase in roughness of the five materials after reciprocal sliding tests under different setups.

Material	Increase in Roughness, ΔRa [%]					
	Initial Ra [µm]	Setup 1	Setup 2	Setup 3	Setup 4	Setup 5
Ti(C,N)-FeNi	0.50 ± 0.06	128	136	170	144	174
Ti(C,N)-FeNiCr	0.49 ± 0.03	143	145	127	167	124
WC-FeNi	0.35 ± 0.04	106	114	134	134	137
WC-FeNiCr	0.36 ± 0.03	125	139	150	139	144
WC-Co	0.33 ± 0.04	121	142	151	145	158

**Table 7:** Steady-state coefficient of friction of the materials after reciprocal sliding tests under different setups.

Material	COF				
	Setup 1	Setup 2	Setup 3	Setup 4	Setup 5
Ti(C,N)-FeNi	0.50	0.42	0.44	0.41	0.48
Ti(C,N)-FeNiCr	0.49	0.43	0.48	0.48	0.50
WC-FeNi	0.33	0.30	0.25	0.29	0.31
WC-FeNiCr	0.41	0.29	0.26	0.29	0.32
WC-Co	0.35	0.33	0.30	0.30	0.28

**Table 8:** Width of wear tracks measured after reciprocal sliding tests under different setups.

Material	Width of Wear Track [µm]				
	Setup 1	Setup 2	Setup 3	Setup 4	Setup 5
Ti(C,N)-FeNi	206 ± 2	214 ± 2	348 ± 7	243 ± 25	323 ± 20
Ti(C,N)-FeNiCr	197 ± 8	206 ± 9	344 ± 26	231 ± 17	309 ± 14
WC-FeNi	132 ± 6	182 ± 5	227 ± 9	214 ± 24	271 ± 35
WC-FeNiCr	144 ± 14	195 ± 9	239 ± 5	193 ± 6	249 ± 3
WC-Co	99 ± 5	192 ± 26	225 ± 26	200 ± 23	223 ± 19

**Table 9:** Calculated wear rates after reciprocal sliding tests under different setups.

Material	Wear Rate [10^−8^ mm^3^/mm]				
	Setup 1	Setup 2	Setup 3	Setup 4	Setup 5
Ti(C,N)-FeNi	2.10 ± 0.29	6.42 ± 1.15	3.79 ± 0.18	6.73 ± 2.58	3.20 ± 0.84
Ti(C,N)-FeNiCr	1.98 ± 0.09	3.48 ± 0.23	3.45 ± 0.90	3.05 ± 0.26	2.96 ± 0.63
WC-FeNi	1.19 ± 0.04	1.85 ± 0.25	0.56 ± 0.03	2.38 ± 0.08	0.56 ± 0.03
WC-FeNiCr	1.34 ± 0.07	1.83 ± 0.05	0.62 ± 0.05	2.24 ± 0.39	0.50 ± 0.01
WC-Co	1.74 ± 0.09	2.32 ± 0.09	0.50 ± 0.15	2.70 ± 0.05	0.49 ± 0.01

**Table 10:** EDS results from the different areas indicated in Figure 7.

Area	Element [wt.%]								
	Ti	W	C	N	Fe	Ni	Cr	O	Co
1	37.4	2.4	5.6	3.7	9.8	2.3	1.3	37.5	-
2	-	67.4	2.0	-	7.7	2.2	1.6	17.4	1.7
3	-	77.4	9.2	-	9.2	1.9	1.5	0.1	0.7

**Table 11:** Values of the exponential coefficient and oxidation rate constant from the fitting curve of the oxidation kinetics of each material at the studied temperatures.

Material	Exponential Coefficient, *n* (R^2^)		Oxidation Rate Constant, *k_n_* [mg^n^/cm^2n^·s]	
	650 °C	800 °C	650 °C	800 °C
Ti(C,N)-FeNi	0.4327 (0.9964)	0.6013 (0.9998)	0.1444	1.9114
Ti(C,N)-FeNiCr	0.6111 (0.9992)	0.5530 (0.9977)	0.1279	2.3308
WC-FeNi	0.3463 (0.9915)	-	5.6042	-
WC-FeNiCr	0.2782 (0.9995)	-	4.5694	-
WC-Co	0.2976 (0.9886)	-	7.3862	-

## Data Availability

The original contributions presented in the study are included in the article, further inquiries can be directed to the corresponding author.
